# Novel Pathogenic Variants in a Cassette Exon of *CCM2* in Patients With Cerebral Cavernous Malformations

**DOI:** 10.3389/fneur.2019.01219

**Published:** 2019-11-20

**Authors:** Christiane D. Much, Konrad Schwefel, Dariush Skowronek, Loay Shoubash, Felix von Podewils, Miriam Elbracht, Stefanie Spiegler, Ingo Kurth, Agnes Flöel, Henry W. S. Schroeder, Ute Felbor, Matthias Rath

**Affiliations:** ^1^Department of Human Genetics, University Medicine Greifswald and Interfaculty Institute for Genetics and Functional Genomics, University of Greifswald, Greifswald, Germany; ^2^Department of Neurosurgery, University Medicine Greifswald, Greifswald, Germany; ^3^Department of Neurology, University Medicine Greifswald, Greifswald, Germany; ^4^Institute of Human Genetics, Medical Faculty, RWTH Aachen University, Aachen, Germany

**Keywords:** cerebral cavernous malformations, novel *CCM2* mutations, *CCM2* transcript analyses, CNV analyses, seizures, cerebral hemorrhage

## Abstract

Autosomal dominant cerebral cavernous malformation (CCM) represents a genetic disorder with a high mutation detection rate given that stringent inclusion criteria are used and copy number variation analyses are part of the diagnostic workflow. Pathogenic variants in either *CCM1* (*KRIT1*), *CCM2* or *CCM3* (*PDCD10*) can be identified in 87–98% of CCM families with at least two affected individuals. However, the interpretation of novel sequence variants in the 5′-region of *CCM2* remains challenging as there are various alternatively spliced transcripts and different transcription start sites. Comprehensive genetic and clinical data of CCM2 patients with variants in cassette exons that are either skipped or included into alternative *CCM2* transcripts in the splicing process can significantly facilitate clinical variant interpretation. We here report novel pathogenic *CCM2* variants in exon 3 and the adjacent donor splice site, describe the natural history of CCM disease in mutation carriers and provide further evidence for the classification of the amino acids encoded by the nucleotides of this cassette exon as a critical region within CCM2. Finally, we illustrate the advantage of a combined single nucleotide and copy number variation detection approach in NGS-based *CCM1*/*CCM2*/*CCM3* gene panel analyses which can significantly reduce diagnostic turnaround time.

## Introduction

Cerebral cavernous malformations (CCMs; MIM: 116860, 603284, 603285) are mulberry-like vascular lesions that are formed by thin-walled and densely packed endothelial channels (1, 2). These low-flow sinusoidal convolutes are characterized by an impaired blood-brain-barrier due to a complex dysfunction of the lining endothelial cells. As a result of recurrent bleeding events, CCM patients often present with non-specific headaches, seizures and stroke-like symptoms (3). Over the last years, however, CCMs have increasingly been identified as incidental findings due to the widespread use of high-resolution magnetic resonance brain imaging (4). Since targeted therapies have not yet been approved, conservative treatment still focuses on the control of CCM-related complications. Surgical resection can be an option for easily accessible symptomatic CCMs, cavernous lesions that cause epilepsy or for deep CCMs that are either symptomatic or have already led to intracerebral hemorrhage. Treatment of CCM patients with brainstem CCMs is often difficult and the risk of post-operative morbidity and mortality always needs to be critically discussed before the indication for surgical resection is made (4).

CCMs may occur sporadically or in an autosomal dominant familial form. The prevalence of symptomatic hereditary CCM has been estimated to be in the range of 1:5,400 to 1:6,200 in the general population (5). Heterozygous germline loss-of-function variants in one of the three genes *CCM1/KRIT1* (MIM 604214), *CCM2/OSM/Malcavernin* (MIM 607929), or *CCM3/PDCD10* (MIM 609118) can be found in the vast majority of familial CCM cases (5). The identification of a pathogenic variant is not only important for the index case but also crucial for genetic counseling of at-risk family members. The mutation detection rate of current molecular genetic *CCM1*/*CCM2*/*CCM3* analyses is 87–98% for families with two or more CCM patients and up to 57–75% for index cases with multiple CCMs but a negative family history (4–6). However, these high mutation detection rates can only be realized with stringent inclusion criteria and a comprehensive workflow for the detection of single nucleotide variants (SNVs), small indels, and copy-number variants (CNVs).

*CCM2* nonsense, frameshift, and splice mutations can be identified in about 13% of CCM cases that meet the inclusion criteria (6). Furthermore, various *CCM2* CNVs have been reported for sporadic and familial CCM cases (7–14). One of these-the deletion of exons 2 to 10 of *CCM2* (LRG_664t2)-is actually a founder mutation in the US population (10, 11). The multiplex ligation-dependent probe amplification (MLPA) technique has long been used as gold standard for copy number quantification but the implementation of gene panel analyses has opened new perspectives for detection of CNVs from NGS data.

In this study, we report two novel and one previously published variants within a cassette exon of *CCM2* and provide evidence for their pathogenicity. In addition, we describe the natural history of CCM disease in mutation carriers and illustrate the advantages of a combined NGS-based SNV/CNV detection workflow in *CCM1*/*CCM2*/*CCM3* gene panel analyses.

## Materials and Methods

### Genetic Analyses

Genetic analyses were performed with written informed consent of all study participants according to the German Gene Diagnostics Act and approval of the local ethics committee of the University Medicine Greifswald (No. B119/10). Genomic DNA was isolated from peripheral blood lymphocytes using the NucleoSpin Blood L Kit (Macherey-Nagel, Düren, Germany). Thirty-one probands were analyzed for pathogenic *CCM1, CCM2*, or *CCM3* variants by hybridization capture-based target enrichment and next generation sequencing and another 2 patients were analyzed by Sanger sequencing as described previously (6). All coding exons and exon-intron-boundaries (± 20 bp) of *CCM1* (exons 5 to 20 according to reference sequence LRG_650t1), *CCM2* (exons 1 to 10 according to LRG_664t2 = exons 1 and 3 to 11 according to LRG_664), and *CCM3* (exons 3 to 9 according to LRG_651t1) were defined as target regions in both approaches. Sequencing libraries were prepared with Nextera Rapid Capture (Panel ID: 113402, Illumina, San Diego, USA) or Agilent SureSelect^QXT^ (Panel ID: 3152261, Agilent Technologies, Santa Clara, USA) custom enrichment kits according to the manufacturers' instructions and sequenced on a MiSeq instrument with 2 × 150 cycles (Illumina, San Diego, USA). FASTQ files were generated with the MiSeq reporter software v2.6.2 (MSR; Illumina, San Diego, USA). The SeqNext module of the Sequence Pilot software (v5.0.0, JSI Medical Systems, Ettenheim, Germany) was used for read mapping, alignment and variant calling. Sanger sequencing data were analyzed with the SeqPatient module of the Sequence Pilot software. The Human Splicing Finder 3.1 (http://www.umd.be/HSF3/), Berkeley Drosophila Genome Project (http://www.fruitfly.org/), NetGene2 (http://www.cbs.dtu.dk/services/NetGene2/), MaxEntScan (http://hollywood.mit.edu/burgelab/maxent/Xmaxentscan_scoreseq.html), and ASSP (http://wangcomputing.com/assp/) tools were used for *in silico* splice predictions.

### CNV Analyses

The SeqNext module (v5.0.0, JSI Medical Systems, Ettenheim, Germany) was used for CNV analyses in a read-depth approach based on a previous report for other target genes (15). In brief, all exons of *CCM1, CCM2*, and *CCM3*, as well as 17 control fragments located on different chromosomes were defined as regions of interest (ROIs). Sequencing data were mapped and aligned to the target region with the SeqNext module. In a first normalization step, the specific read depth of each target exon and the average read depth of all control fragments were used to calculate the relative product coverage (RPC) for each *CCM1, CCM2*, and *CCM3* exon. In addition to the patient sample, at least five control samples from the same sequencing run were analyzed in the same way. In a next step, each RPC of the patient sample was normalized to the corresponding average reference RPC that had been calculated from all control samples. Thresholds of ≤ 70 and ≥ 130% were used for deletion and duplication calling, respectively. The Salsa MLPA Kits P130-A3 and P131-B1 were used according to the manufacturer's instructions to validate the *CCM1, CCM2*, and *CCM3* deletions (MRC-Holland, Amsterdam, The Netherlands).

### *CCM2* Transcript Analyses

RNA was isolated from peripheral blood lymphocytes using the PAXgene Blood RNA Kit (PreAnalytiX, Hombrechtikon, Switzerland). Three hundred nanogram of total RNA were reverse described into cDNA using SuperScript™ III Reverse Transcriptase (Thermo Fisher Scientific, Waltham, USA). The exons 1, 3, 4, and 5 (exon numbering according to LRG_664) of the *CCM2* transcript LRG_664t2 were amplified using specific forward (5′-GCGGCGATATGGAAGAGG-3′) and reverse (5'-GCACCCTGAGGATGATATC-3′) primers (7, 16). PCR products were size-separated by agarose gel electrophoresis and visualized on a Gel Doc™ EZ Imager (Bio-Rad, Hercules, USA). The excised fragments were purified with the Zymoclean™ Gel DNA Recovery Kit (Zymo Research, Freiburg, Germany) and analyzed by Sanger sequencing.

## Results

In our present study, we included 33 CCM index cases with multiple CCMs and/or a positive family history that had been analyzed for pathogenic variants in *CCM1, CCM2* or *CCM3* between 2017 and 2019 (Table 1). As expected, most patients with an autosomal dominant CCM disease were heterozygous for a pathogenic or likely pathogenic *CCM1* variant (*n* = 18) (Table S1). Another four patients had pathogenic *CCM2* germline variants and two patients were *CCM3* mutation carriers (Table S1). The unexpected observation that three of the four *CCM2* mutations clustered in a cassette exon that can be skipped in the splicing process prompted us to focus on these *CCM2* variants that had been identified in families 1–3 in more detail (Table 2).

**Table 1:** Overview of the CCM cohort.

	***n* = 33**
Sex: M/F	16/17
Mean age ± standard deviation in years (range)	32.6 ± 18.1 (1–67)
**Clinical manifestation**	
Single/multiple CCM	4/29 (12.1%/87.9%)
Cerebral hemorrhage	6 (18.2%)
Seizures	6 (18.2%)
Positive family history^#^	17/33 (51.5%)
Overall mutation detection rate	24/33 (72.7%)
Mutation detection rate for patients with a positive familial history	16/17 (94%)
Mutation detection rate for patients with a negative family history	8/16 (50%)
**Genotypes**	
Pathogenic *CCM1* variant	18
Pathogenic *CCM2* variant	4
Pathogenic *CCM3* variant	2

# *Since neuroimaging data were not available for some relatives of the CCM index cases, the classification as familial CCM case is based on clinical symptoms of at-risk family members that were suggestive of CCM disease (e.g., seizures, hemorrhagic stroke)*.

**Table 2:** Overview of the pathogenic *CCM2* SNVs and indel variants reported in our present study.

**Family number**	**Exon (LRG_664)**	**Exon (LRG_664t2)**	**Nucleotide change**	**Amino acid change**	**Mutation type**	**References**
1	Ex 3	Ex 2	c.204+1G>A	p.(Pro11_Lys68del)	Splice site variant	NOVEL
2	Ex 3	Ex 2	c.169dupA	p.(Arg57Lysfs*8)	Frameshift variant	NOVEL
3	Ex 3	Ex 2	c.134_135delTG	p.(Val45Glyfs*6)	Frameshift variant	(17)

### Clinical Data of Families 1–3

**Figure 1:**
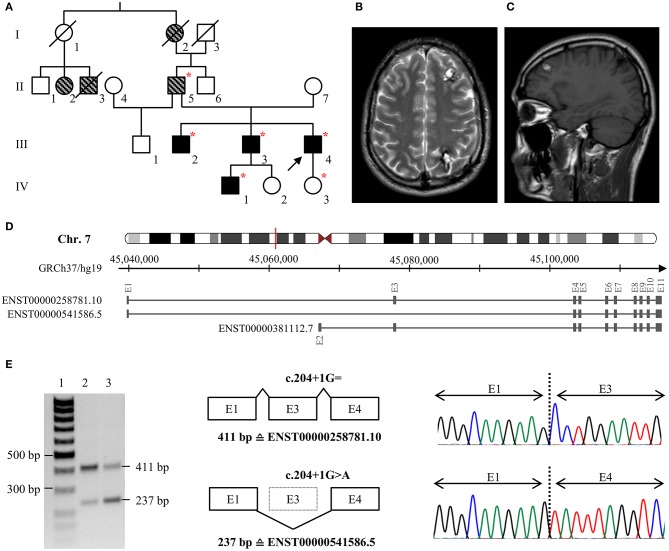
**(A)** Pedigree of family 1. The index case (III/4) is marked with an arrow. Black filled symbols represent patients with CCMs, gray striped symbols indicate relatives with neurological symptoms that are suggestive for CCM. Deceased family members are crossed out. Red stars indicate heterozygous carriers of the pathogenic *CCM2* splice variant c.204+1G>A. **(B,C)** Representative axial T2-weighted **(B)** and sagittal T1-weighted **(C)** MR images of the index case at the age of 32. **(D)** Schematic exon-intron structure of *CCM2* and selected protein-coding *CCM2* transcripts that are listed in the ENSEMBL database. ENST00000258781.10 and ENST00000541586.5 are both expressed in blood lymphocytes but ENST00000541586.5 in which exon 3 is skipped is less abundant in brain or blood vessels. **(E)** Skipping of exon 3 on the c.204+1G>A *CCM2* allele was confirmed by RT-PCR and cDNA sequencing. Lane 1: size marker, lane 2: control sample, lane 3: patient III/4. E, exon.

The index patient of family 1 (III/4, Figure 1A) was first admitted to neurosurgery at the age of 25. He reported a 6-year history of headaches and focal seizures with intermittent numbness and tingling in his right arm and leg. Magnetic resonance imaging (MRI) identified two small CCMs in his left frontal and right occipital lobe as well as a large cavernoma in his left parietal lobe (20 × 18 × 20 mm). The latter was resected by minimally invasive microsurgery. Histological evaluation confirmed CCM diagnosis. At the age of 28, the frequency of the index patient's seizures increased again, and the anti-epileptic therapy was intensified. However, it was not possible to prevent seizures with levetiracetam at maximum dosage of 2 × 1,500 mg. Since an increase in the size of the left frontal CCM (Figures 1B,C) was noticed, it was finally resected by neurosurgery at the age of 33. At last follow-up at the age of 35 years, he still reported epileptic seizures and two novel CCMs were identified in his right frontal lobe and cerebellum. Anti-epileptic therapy was modified to a combination of brivaracetam, lacosamide, perampanel, and eslicarbazepine acetate.

Patient III/4 reported a positive family history for CCMs. His eldest brother (III/2) has had trigeminal neuralgia since the age of 23, and six cavernous lesions were identified on his MRI. The index patient's second brother (III/3) became symptomatic with three focal to bilateral tonic-clonic seizures at the age of 32. One cavernous lesion of 25 × 25 × 20 mm in his left frontal lobe demonstrated signs of an acute hemorrhage and was resected by neurosurgery. In addition, at least seven small CCMs were found in both hemispheres. Two cavernous malformations were also identified in his yet asymptomatic 10-year old son (IV/1) at the age of five. The index patient's father (II/5) had a stroke at the age of 33 and reported chronic headaches. Because of his pacemaker, no MRI data were available for him. Furthermore, the index patient's paternal grandmother (I/2) had chronic headaches, and two cousins of his father (II/2, II/3) had seizures.

**Figure 2:**
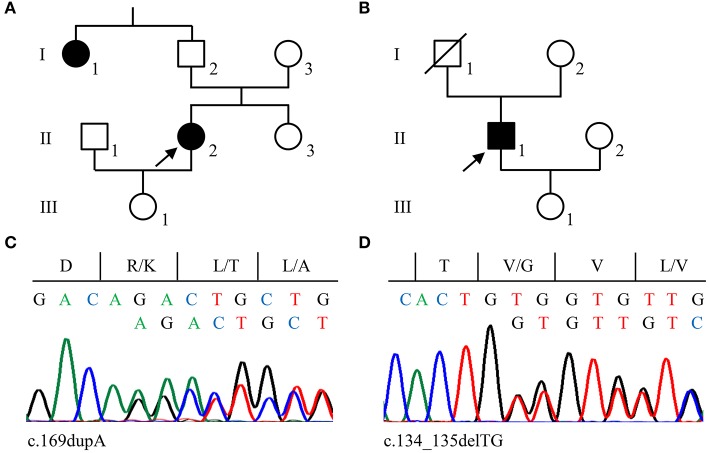
Pedigrees of families 2 **(A)** and 3 **(B)**. Arrows indicate the index cases in each family. **(C,D)** Sanger sequencing validation of pathogenic *CCM2* variants identified in family 2 (c.169dupA; C) and family 3 (c.134_135delTG; D). Nucleotide and protein sequences are shown above each chromatogram.

The 46-year-old female index patient of family 2 (II/2, Figure 2A) reported a 1^1^/_2_-year history of chronic headaches and recurrent attacks of vertigo that had lasted up to 1 min. MRI identified two CCMs located in her cerebellum and frontal lobe. Since her paternal aunt (I/1, Figure 2A) had a CCM bleeding, she was classified as familial CCM case. The male index patient of family 3 (II/1, Figure 2B) had five CCMs located in both hemispheres and became symptomatic at the age of 30 with focal to bilateral tonic-clonic seizures. A sufficient seizure control could be achieved by levetiracetam monotherapy. The patient appears to be a sporadic CCM case since no neurological symptoms that would have been indicative for a CCM disease were reported for his maternal and paternal lineage. However, a cryptic familial case cannot be excluded since his father died of colorectal cancer at the age of 75 without molecular genetic analysis and the index patient's 71-year-old asymptomatic mother decided against predictive genetic testing or MRI.

### Genetic Analyses for Families 1–3

A novel substitution at position +1 of the highly conserved donor splice site of exon 3 was identified in family 1 [LRG_664t2: c.204+1G>A]. *In silico* predictions indicated disruption of normal *CCM2* splicing. Therefore, we decided to perform additional transcript analyses. RT-PCR demonstrated skipping of exon 3 on the mutant *CCM2* allele (Figures 1D,E). Moreover, a heterozygous SNP in exon 3 that was located *in cis* with the *CCM2* splice donor variant could not be identified by cDNA sequencing indicating complete exon skipping. The loss of exon 3 leads to an in-frame deletion of 58 amino acids on protein level [LRG_664p2: p.(Pro11_Lys68del)]. It is noteworthy to mention that this alteration involves the first ten amino acids of the PTB domain of CCM2. Targeted *CCM2* sequence analyses of blood samples from multiple affected family members (III/2; III/3; II/5; IV/1) demonstrated co-segregation of the splice site variant with CCM disease in family 1. According to the latest ACMG guidelines for the interpretation of sequence variants (18), the splice site variant was classified as pathogenic.

*CCM2* loss-of-function variants were also found in families 2 and 3. A novel pathogenic 1-bp duplication [LRG_664t2: c.169dupA; LRG_664p2: p.(Arg57Lysfs^*^8)] was detected in the blood sample of the index patient of family 2 (Figure 2C) and a 2-bp deletion [LRG_664t2: c.134_135delTG; LRG_664p2: p.(Val45Glyfs^*^6)] was identified in family 3 (Figure 2D). The latter had previously been described in an Italian CCM family (17) and was listed as likely pathogenic in the ClinVar database (ClinVar-ID: 590648). Unfortunately, a blood sample from the maternal aunt of the index patient in family 2 was not available to test for the *CCM2* variant. Of note, neither the two frameshift variants in exon 3 nor the *CCM2* splice site variant that had been identified in family 1 are listed in the Genome Aggregation Database (gnomAD) which contains 125,748 whole-exome and 15,708 whole-genome datasets.

### CNV Detection From NGS Data

**Figure 3:**
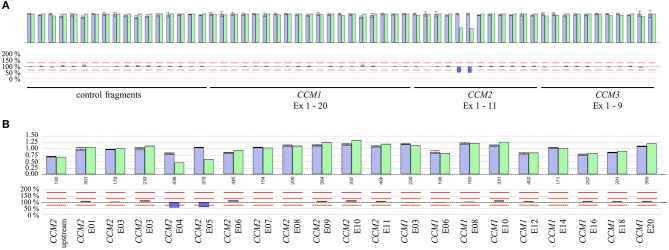
**(A)** Identification of a deletion of exons 4 and 5 of the *CCM2* gene by NGS-based CNV analysis. The ROIs and their relative product coverages are shown in the upper histogram for control samples (blue) and the sample of the CCM proband (green). The ratios for the relative coverage of each ROI are shown in the lower panel. Red lines indicate the detection threshold for deletions and duplications. As some exons of *CCM1-3* have been joined to combined ROIs and alternative exons are also included in the CNV analysis, the number of analyzed ROIs is not equal to the number of exons of *CCM1-3*. **(B)** Verification of the heterozygous *CCM2* deletion by MLPA analysis.

Besides SNVs and small indels that can be found in exon 3 of *CCM2*, a complete in-frame deletion of this exon has been reported in the literature (10, 16). Therefore, we analyzed the available NGS data of the CCM index cases of our cohort in a combined workflow for SNVs and CNVs. Notably, we did not detect a deletion of exon 3 of *CCM2* but three other CNVs in 31 evaluated samples. In particular, we found a heterozygous deletion of the exons 4 and 5 of *CCM2* (exon numbering according to LRG_664) in a 40-year-old symptomatic CCM patient (Figures 3A,B). The relative product coverage ratios (RPC ratios) of both exons were reduced to 52 and 49%, respectively. In addition, heterozygous deletions of the exons 1 to 6 of *CCM1* and of the exons 8 and 9 of the *CCM3* gene (5) were detected in two other CCM patients. RPC ratios were reduced to 49–63 and 41–43%, respectively (Figures S1A-C). All three CNVs were verified by MLPA analysis. Our data demonstrate that apparently mutation-negative CCM patients always have to be analyzed for CNVs and that a combined NGS-based SNV/CNV detection workflow can reduce the diagnostic turnaround time since it renders time-consuming MLPA analyses unnecessary.

## Discussion

We here present comprehensive clinical and genetic data of three CCM2 families with pathogenic variants in a cassette exon of *CCM2*, provide further evidence for the classification of exon 3 as a critical region within *CCM2*, and describe our procedure for NGS-based CNV analyses.

Familial CCM can have many clinical faces. Pathogenic *CCM1, CCM2*, and *CCM3* variants are associated with incomplete and locus-specific penetrance of 63, 55, and 68%, respectively (19). In addition, expressivity even between family members sharing the same pathogenic variant can be extremely variable. *CCM2* mutation carriers reported in this study mainly became symptomatic in their third to fifth decade of life. The mean age at first clinical symptoms of the four symptomatic *CCM2* mutation carriers of family 1 (II/5; III/2; III/3; III/4) and the two index patients of family 2 and 3 was 30.2 years (range 19–44). Hence, it was slightly lower than that described for other CCM2 patients before. The mean age of onset reported by Denier and colleagues was 34.9 years (range 1–69) (19) and our group has observed a mean age at referral to genetic testing of 40.8 years (range 16–71) (6). The mean number of cavernous lesions in our symptomatic CCM cases was also slightly lower (mean number: 5.3; range 2 to 8) than the mean number of 6.6 CCM lesions identified on T2-weighted MRIs of 37 other *CCM2* mutation carriers described in the literature (19).

The identification of a pathogenic variant within a CCM family allows predictive testing for at-risk family members. The exclusion of a known familial variant relieves relatives of anxieties and renders special clinical surveillance unnecessary. However, interpretation of novel *CCM2* variants-especially of those in exon 3-can be challenging since there are several alternatively spliced *CCM2* transcripts. The full-length CCM2 protein with its 444 amino acids has two major functional domains. While the phosphotyrosine binding (PTB) domain is thought to be essential for binding to CCM1 (20), the function of the harmonin-homology domain (HHD) at the C-terminus has not yet been fully elucidated. However, another *CCM2* transcript (ENST00000541586.5, Figure 1D) without exon 3, which encodes for part of the PTB domain, is highly expressed in blood lymphocytes and spleen but less abundant in brain or blood vessels. Of note, *in vitro* studies have demonstrated that deletion of the amino acids encoded by exon 3 abrogates formation of a ternary CCM1/CCM2/CCM3 protein complex (16). Thus, the alternative *CCM2* transcript seems to be unable to compensate for loss of the full-length CCM2 protein. To the best of our knowledge, at least ten pathogenic *CCM2* SNVs and small indel variants in exon 3 have been described so far (Table S2). These mutations account for ~15% of all known *CCM2* nonsense, frameshift and splice mutations (HGMD Professional 2019.1). Taken together, these data implicate that only the full-length CCM2 protein but not an alternative isoform without the 58 amino acids encoded by exon 3 can maintain cerebrovascular stability and quiescence in brain endothelial cells. Therefore, exon 3 can be defined to encode for a “well-established functional domain” according to the ACMG guidelines for variant interpretation (18).

Molecular genetic analyses of the *CCM1*/*CCM2*/*CCM3* genes have long been performed in a step-wise approach. Sequencing for identification of SNVs was followed by MLPA analyses for CNV detection (6). CNVs have been defined as duplications and deletions of at least 1 kb and belong-together with inversions of the same size-to structural genome variants (21). In a modern diagnostic sense, single exon deletions and duplications (> 50 bp) are also classified as CNVs (22). More than 60 different single- and multi-exon deletions and duplications in *CCM1, CCM2*, and *CCM3* are currently listed in the Human Gene Mutation Database (HGMD Professional 2019.1). The widespread use of NGS gene panel analyses and the further development of bioinformatic algorithms nowadays allow SNV and CNV detection in a single NGS pipeline. The advantage of such a comprehensive approach is the significant reduction of costs and turnaround time when compared to sequential Sanger sequencing and MLPA analyses. Additionally, NGS-based CNV analyses enable a higher resolution and more precise breakpoint detection (23). However, current gene panel analyses also have limitations. We have recently identified a 24 kb inversion by whole genome sequencing that includes exon 1 of *CCM2* in a family with multiple CCM cases (24). The detection of such copy-neutral structural genomic rearrangements in targeted gene panel approaches is extremely challenging and often even impossible.

In conclusion, the results of our study demonstrate that accurate phenotyping and genotyping are crucial for variant interpretation in *CCM1*/*CCM2*/*CCM3* gene panel sequencing and that CNV calling from NGS data can help to make the analyses more effective.

## Data Availability Statement

All datasets analyzed for this study are included in the article/Supplementary Material.

## Ethics Statement

The studies involving human participants were reviewed and approved by Ethics committee of the University Medicine Greifswald (No. B119/10). Written informed consent of the study participants to publish all data and information that are presented in our current manuscript was obtained.

## Author Contributions

CM, MR, and UF contributed to the intellectual conception and the design of the study. CM, KS, SS, and DS performed most of the experiments. CM, KS, ME, SS, DS, IK, MR, and UF contributed to interpretation of the genetic results. LS, FP, ME, IK, AF, and HS cared for the patients and contributed to interpretation of the clinical and neuroimaging results, CM, UF, and MR drafted the manuscript. All authors contributed to writing.

### Conflict of Interest

The authors declare that the research was conducted in the absence of any commercial or financial relationships that could be construed as a potential conflict of interest.
