# Supplementary material for: Novel Pathogenic Variants in a Cassette Exon of CCM2 in Patients With Cerebral Cavernous Malformations
Source: Front Neurol. 2019 Nov 20;10:1219. doi: 10.3389/fneur.2019.01219 (PMC6879547; doi:10.3389/fneur.2019.01219)
Supplement: Supplementary file 1 [file Data_Sheet_1.PDF]

Supplementary Material

Table S1: Pathogenic and likely pathogenic variants identified in CCM patients

Gene	Nucleotide Change	Amino Acid Change	Number of patients with this variant	Reference
CCM1	c.268C>T	p.(Arg90*)	1	(Cave-Riant et al., 2002)
	c.535C>T	p.(Arg179*)	2	(Cave-Riant et al., 2002)
	c.547_548insAAGTC	p.(Leu183Glnfs*6)	1	novel
	c.730-1G>A	p.?	1	(Davenport et al., 2001)
	c.990delG	p.(Trp330Cysfs*12)	1	novel
	c.1093G>T	p.(Gly365*)	1	(Spiegler et al., 2014)
	c.1278_1281delAATG	p.(Asp428Glyfs*8)	1	(Laberge-le Couteulx et al., 1999)
	c.1363C>T	p.(Gln455*)	1	(Sahoo et al., 1999)
	c.1762dupA	p.(Thr588Asnfs*6)	1	ClinVar ID 407272
	c.1820dupA	p.(Asn607Lysfs*6)	1	(Gabelia et al., 2016)
	c.1961_1962delAA	p.(Lys654Serfs*21)	1	(Spiegler et al., 2014)
	c.2012delA	p.(Asn671Thrfs*36)	1	(Spiegler et al., 2019)
	c.2025+1G>A	p.?	1	(Denier et al., 2004)
	c.2051delG	p.(Gly684Valfs*23)	1	LOVD #0000532977
	c.2069delT	p.(Leu690Trpfs*17)	1	novel
	c.2165delT	p.(Leu722*)	1	novel
	Deletion of exons 1 to 6	p.?	1	(Riant et al., 2013)
CCM2	c.204+1G>A	p.(Pro11_Lys68del)	1	novel
	c.169dupA	p.(Arg57Lysfs*8)	1	novel
	c.134_135delTG	p.(Val45Glyfs*6)	1	(Nardella et al., 2018)
	Deletion of exons 4 and 5	p.?	1	(Riant et al., 2013)
CCM3	c.558-1_558insTTTAG	p.?	1	novel
	Deletion of exons 8 and 9	p.?	1	(Spiegler et al., 2018)

Table S2: Overview of previously reported disease-causing variants in exon 3 of *CCM2*

**Nucleotide Change**	**Amino Acid Change**	**Mutation Type**	**Reference**
c.54_55del	p.(Arg19Serfs*6)	frameshift variant	(Gianfrancesco et al., 2007)
c.55C>T	p.(Arg19*)	nonsense variant	(Verlaan et al., 2004)
c.56del	p.(Arg19Glnfs*4)	frameshift variant	(Liquori et al., 2003)
c.85_86dup	p.(Asp29Glufs*9)	frameshift variant	(D'Angelo et al., 2011)<br>(described as c.82_83insAG; 29fs37X)
c.97del	p.(His33Metfs*4)	frameshift variant	(Huang et al., 2016)
c.113_116del	p.(Glu38Glyfs*21)	frameshift variant	(Jih et al., 2018)
c.134_135del	p.(Val45Glyfs*6)	frameshift variant	(Nardella et al., 2018)
c.169_172del	p.(Arg57Cysfs*2)	frameshift variant	(Liquori et al., 2003)
c.193_204del	p.(Lys65_Lys68del)	in-frame deletion	(Tsutsumi et al., 2013)
c.204+2T>C	p.?	splice site variant	(Belousova et al., 2017)
c.(30+1_31-1)_(204+1_205-1)del	p.(Pro11_Lys68del)	single exon deletion	(Stahl et al., 2008)<br>(Liquori et al., 2007)

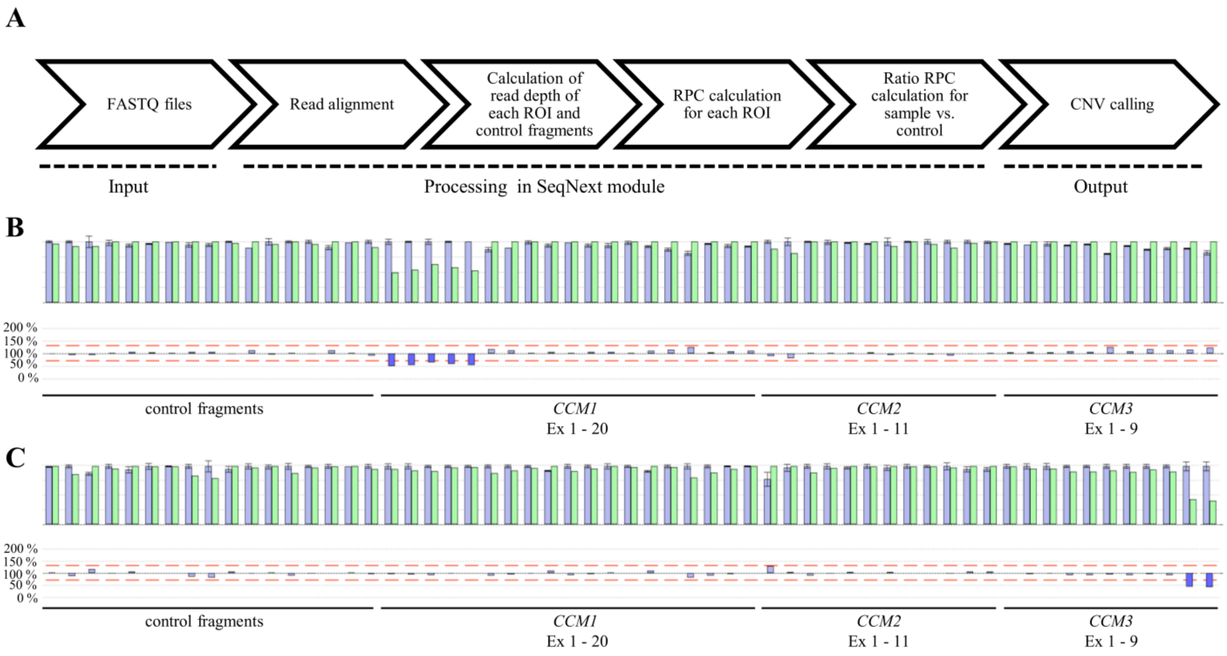

Figure S1: (A) Schematic workflow of the procedure for our NGS-based CNV analysis. (B) Detection of heterozygous deletions of the exons 1 to 6 of the *CCM1* gene and (C) of the exons 8 and 9 of the *CCM3* gene by NGS-based CNV analysis. The latter has previously been reported (Spiegler et al., 2018). Detection thresholds for deletions and duplication calling are marked by red lines. Relative product coverages are highlighted by green (proband) and blue bars (control). Some exons of *CCM1-3* have been joined to combined ROIs (e.g. exons 1 and 2 of *CCM1*) and alternative exons are also included in the CNV analysis. Therefore, the number of analyzed ROIs is not equal to the number of exons of *CCM1-3*.

frontiers

References:

Belousova, O.B., Okishev, D.N., Ignatova, T.M., Balashova, M.S., and Boulygina, E.S. (2017). Hereditary Multiple Cerebral Cavernous Malformations Associated with Wilson Disease and Multiple Lipomatosis. *World Neurosurg* 105**,** 1034 e1031-1034 e1036.

Cave-Riant, F., Denier, C., Labauge, P., Cecillon, M., Maciazek, J., Joutel, A., Laberge-Le Couteulx, S., and Tournier-Lasserve, E. (2002). Spectrum and expression analysis of *KRIT1* mutations in 121 consecutive and unrelated patients with Cerebral Cavernous Malformations. *Eur J Hum Genet* 10**,** 733-740.

D'angelo, R., Marini, V., Rinaldi, C., Origone, P., Dorcaratto, A., Avolio, M., Goitre, L., Forni, M., Capra, V., Alafaci, C., Mareni, C., Garre, C., Bramanti, P., Sidoti, A., Retta, S.F., and Amato, A. (2011). Mutation analysis of *CCM1*, *CCM2* and *CCM3* genes in a cohort of Italian patients with cerebral cavernous malformation. *Brain Pathol* 21**,** 215-224.

Davenport, W.J., Siegel, A.M., Dichgans, J., Drigo, P., Mammi, I., Pereda, P., Wood, N.W., and Rouleau, G.A. (2001). *CCM1* gene mutations in families segregating cerebral cavernous malformations. *Neurology* 56**,** 540-543.

Denier, C., Labauge, P., Brunereau, L., Cave-Riant, F., Marchelli, F., Arnoult, M., Cecillon, M., Maciazek, J., Joutel, A., Tournier-Lasserve, E., Societe Francaise De, N., and Societe De Neurochirurgie De Langue, F. (2004). Clinical features of cerebral cavernous malformations patients with *KRIT1* mutations. *Ann Neurol* 55**,** 213-220.

Gabelia, D., Pikija, S., and Al-Schameri, A.R. (2016). Acute Neck Pain Progressing to Hemiparesis With Brain and Spinal Cord Lesions. *JAMA Neurol* 73**,** 1491-1492.

Gianfrancesco, F., Cannella, M., Martino, T., Maglione, V., Esposito, T., Innocenzi, G., Vitale, E., Liquori, C.L., Marchuk, D.A., and Squitieri, F. (2007). Highly variable penetrance in subjects affected with cavernous cerebral angiomas (CCM) carrying novel *CCM1* and *CCM2* mutations. *Am J Med Genet B Neuropsychiatr Genet* 144B**,** 691-695.

Huang, W.Q., Lu, C.X., Zhang, Y., Yi, K.H., Cai, L.L., Li, M.L., Wang, H., Lin, Q., and Tzeng, C.M. (2016). A Novel *CCM2* Gene Mutation Associated with Familial Cerebral Cavernous Malformation. *Front Aging Neurosci* 8**,** 220.

Jih, K.Y., Chung, C.P., Chang, Y.Y., Hung, P.L., Soong, B.W., Liao, Y.C., Lan, M.Y., and Lee, Y.C. (2018). Mutational analysis of *CCM1*, *CCM2* and *CCM3* in a Han Chinese cohort with multiple cerebral cavernous malformations in Taiwan. *Clin Genet* 94**,** 389-390.

Laberge-Le Couteulx, S., Jung, H.H., Labauge, P., Houtteville, J.P., Lescoat, C., Cecillon, M., Marechal, E., Joutel, A., Bach, J.F., and Tournier-Lasserve, E. (1999). Truncating mutations in *CCM1*, encoding KRIT1, cause hereditary cavernous angiomas. *Nat Genet* 23**,** 189-193.

Liquori, C.L., Berg, M.J., Siegel, A.M., Huang, E., Zawistowski, J.S., Stoffer, T., Verlaan, D., Balogun, F., Hughes, L., Leedom, T.P., Plummer, N.W., Cannella, M., Maglione, V., Squitieri, F., Johnson, E.W., Rouleau, G.A., Ptacek, L., and Marchuk, D.A. (2003). Mutations in a gene encoding a novel protein containing a phosphotyrosine-binding domain cause type 2 cerebral cavernous malformations. *Am J Hum Genet* 73**,** 1459-1464.

Liquori, C.L., Berg, M.J., Squitieri, F., Leedom, T.P., Ptacek, L., Johnson, E.W., and Marchuk, D.A. (2007). Deletions in *CCM2* are a common cause of cerebral cavernous malformations. *Am J Hum Genet* 80**,** 69-75.

Nardella, G., Visci, G., Guarnieri, V., Castellana, S., Biagini, T., Bisceglia, L., Palumbo, O., Trivisano, M., Vaira, C., Scerrati, M., Debrasi, D., D'angelo, V., Carella, M., Merla, G., Mazza, T., Castori, M., D'agruma, L., and Fusco, C. (2018). A single-center study on 140 patients with cerebral cavernous malformations: 28 new pathogenic variants and functional characterization of a *PDCD10* large deletion. *Hum Mutat* 39**,** 1885-1900.

Riant, F., Cecillon, M., Saugier-Veber, P., and Tournier-Lasserve, E. (2013). CCM molecular screening in a diagnosis context: novel unclassified variants leading to abnormal splicing and importance of large deletions. *Neurogenetics* 14**,** 133-141.

Sahoo, T., Johnson, E.W., Thomas, J.W., Kuehl, P.M., Jones, T.L., Dokken, C.G., Touchman, J.W., Gallione, C.J., Lee-Lin, S.Q., Kosofsky, B., Kurth, J.H., Louis, D.N., Mettler, G., Morrison, L., Gil-Nagel, A., Rich, S.S., Zabramski, J.M., Boguski, M.S., Green, E.D., and Marchuk, D.A. (1999). Mutations in the gene encoding KRIT1, a Krev-1/rap1a binding protein, cause cerebral cavernous malformations (CCM1). *Hum Mol Genet* 8**,** 2325-2333.

Spiegler, S., Najm, J., Liu, J., Gkalympoudis, S., Schroder, W., Borck, G., Brockmann, K., Elbracht, M., Fauth, C., Ferbert, A., Freudenberg, L., Grasshoff, U., Hellenbroich, Y., Henn, W., Hoffjan, S., Huning, I., Korenke, G.C., Kroisel, P.M., Kunstmann, E., Mair, M., Munk-Schulenburg, S., Nikoubashman, O., Pauli, S., Rudnik-Schoneborn, S., Sudholt, I., Sure, U., Tinschert, S., Wiednig, M., Zoll, B., Ginsberg, M.H., and Felbor, U. (2014). High mutation detection rates in cerebral cavernous malformation upon stringent inclusion criteria: one-third of probands are minors. *Mol Genet Genomic Med* 2**,** 176-185.

Spiegler, S., Rath, M., Much, C.D., Sendtner, B.S., and Felbor, U. (2019). Precise *CCM1* gene correction and inactivation in patient-derived endothelial cells: Modeling Knudson's two-hit hypothesis in vitro. *Mol Genet Genomic Med* 7**,** e00755.

Spiegler, S., Rath, M., Paperlein, C., and Felbor, U. (2018). Cerebral Cavernous Malformations: An Update on Prevalence, Molecular Genetic Analyses, and Genetic Counselling. *Mol Syndromol* 9**,** 60-69.

Stahl, S., Gaetzner, S., Voss, K., Brackertz, B., Schleider, E., Surucu, O., Kunze, E., Netzer, C., Korenke, C., Finckh, U., Habek, M., Poljakovic, Z., Elbracht, M., Rudnik-Schoneborn, S., Bertalanffy, H., Sure, U., and Felbor, U. (2008). Novel *CCM1*, *CCM2*, and *CCM3* mutations in patients with cerebral cavernous malformations: in-frame deletion in CCM2 prevents formation of a CCM1/CCM2/CCM3 protein complex. *Hum Mutat* 29**,** 709-717.

Tsutsumi, S., Ogino, I., Miyajima, M., Ikeda, T., Shindo, N., Yasumoto, Y., Ito, M., and Arai, H. (2013). Genomic causes of multiple cerebral cavernous malformations in a Japanese population. *J Clin Neurosci* 20**,** 667-669.

Verlaan, D.J., Laurent, S.B., Rochefort, D.L., Liquori, C.L., Marchuk, D.A., Siegel, A.M., and Rouleau, G.A. (2004). *CCM2* mutations account for 13% of cases in a large collection of kindreds with hereditary cavernous malformations. *Ann Neurol* 55**,** 757-758.
